# Editorial

**Published:** 1865-02

**Authors:** 


### Editorial.

---

NOTICE.—Our readers are all aware that during the last two years the prices of labor and paper have more than doubled. And by an estimate just given to us by our printer, we find that the actual cost of each number of the EXAMINER when ready for the mail is twenty-three cents; or *two dollars and seventy-six cents* for the twelve numbers to which each subscriber is entitled during the year.

This makes it necessary either to reduce the size of the EXAMINER or increase its price. Believing that the great majority of our subscribers would prefer the latter, we shall from this date make the subscription price *three dollars* per annum. All the subscribers who have already paid in advance for 1865 will receive their numbers to the end of the year without additional charge. But all new subscribers, and those now on our books, who have not paid for the present year, will be charged *three dollars* per annum from the first of January, 1865. Owing to an accidental circumstance in the printing office, the publication of the EXAMINER for December, 1864, was delayed several weeks. We shall soon regain the lost time, however, and keep the issue at its proper date.

CHICAGO MEDICAL COLLEGE (*Medical Department of Lake Forest University*).—The Sixth Annual Commencement for the conferring of degrees, in this institution, took place on the 7th of March, 1865. The lecture-room of the college was closely crowded with an intelligent audience. The exercises were opened by prayer by the Rev. Dr. KITCHELL. In the absence of Prof. JOHNSON, president of the faculty, Prof. N. S. DAVIS officiated as president, and after some remarks in relation to the history and progress of the College, those members of the Junior Class, who had passed their examinations, were called up and publicly presented with their Certificates of progress, accompanied by an earnest charge to so continue their studies, as not only to master the facts and principles of the medical science, but also to acquire a mental discipline and development capable of using those facts and principles with the highest degree of success. The list of Candidates for Graudation was then called by the Secretary, and the Degree of Doctor of Medicine was conferred, by the Acting President, on twenty-six young men, accompanied by an impressive charge in relative to the nature of their profession, the responsibilities it imposes, and the necessity of unbending virtue and unremitting labor and study, as the only reliable means for professional success. The President also announced that the ad-eundem Degree of M.D., had been conferred on four candidates, and the Honorary Degree on one; making the whole number of graduates *thirty-one.* The Committee on the awarding of Prizes, reported that the prize for the best Inaugural Thesis had been awarded to John C. Pratt; the prize for the second best Inaugural Thesis, to Henry P. Merriman; and the prize for having sustained the best examination for graduation, to Melvin N. Rust.

The Valedictory Address was then delivered by Prof. J. S. JEWELL. The Address was highly appropriate for the occasion, and admirably calculated to benefit those to whom it was addressed. The subject was, the necessity of mental discipline, and the study of metaphysics and logic as a means of obtaining it. The names of the graduates are as follows:—

| | |
|---|---|
| H. C. Barrell,--------U.S.A. | S. McGiffin,------------Ohio. |
| M. Block,------------Illinois. | H. P. Merriman,-----Illinois. |
| R. F. Blount, -------Indiana. | R. C. Moore,--------- " |
| J. V. Campbell,-----Illinois. | S. M. Pegram,-------- " |
| Thomas Cochran,---- " | W. H. Pevler, -------- " |
| Daniel Duckett,------ " | J. C. Pratt, -------Michigan. |
| J. F. Fleming,------- " | M. N. Rust,----------Illinois. |
| J. W. Frazey, ------- " | W. D. Saxton,----Wisconsin. |
| A. Grætinger, ----Wisconsin. | W. H. Searles,---- " |
| A. B. Hanna,--------Illinois. | J. S. Sherman, -----Illinois. |
| Charles Isham, ------- " | C. M. Spalding,------- " |
| G. A. Kuechen, --------Iowa. | J. W. St. John, ---Wisconsin. |
| J. E. Link, ----------Illinois. | J. F. Williams, ------Illinois. |

*Ad-eundem Degree.*

| | |
|---|---|
| David Cole, -------------Iowa. | J. E. Thayer,------Wisconsin. |
| D. Hinckley, ----------Illinois. | D. B. Wren,------------Ohio. |

*Honorary Degree.*

E. C. DePuy, ---------------------------Illinois.

The whole number of matriculants, for the Term just closed, was ninty-seven. It will thus be seen, that the Chicago Medical College is steadily advancing in prosperity and usefulness. The next Summer Reading and Clinical Term commences on the second Monday in March, and continues until the fourth of July. For particular information in relation to any matter connected with the College, application may be made to the Secretary of the Faculty, Prof. E. Andrews, 101 Washington Street, Chicago.

---

Chicago Medical Society.—At the regular weekly meeting of the Chicago Medical Society, on the evening of March 11th, 1865, Dr. Hildreth, Surgeon to the Desmarres Hospital in this city, for the treatment of soldiers affected with diseases of the eye and ear, presented to the Society a soldier, about 20 years of age, with a striking want of development in his external auditory apparatus. Both external ears were very small and imperfect in their formation, and the external auditory canals or meatus were entirely *absent*, both in the soft parts and the temporal bone. Yet the individual could hear ordinary conversation without any difficulty. The defect was congenital. During the discussion to which this case gave rise, Dr. HILDRETH mentioned the case of a soldier who had become deaf, from supposed anchylosis of the small bones of the ear, but whose hearing was restored by a severe concussion. And Dr. DAVIS alluded to a case, which had been reported to him about two years since, of congenital deafness; the individual having been educated as a deaf mute; but in which hearing was restored during the progress of an attack of severe *variola*, occurring after the patient had attained the age of 45 years.

The Society spent the remainder of the evening in an interesting and profitable discussion on the nature and treatment of erysipelas.

---

MORTALITY OF CHICAGO FOR FEBRUARY, 1865.—The following table of mortality is condensed from the Health Officer's report. The month was characterized by a prevalence of dry, pleasant, and only moderately cold weather:—

DISEASES.

| | | | |
|---|---|---|---|
| Accidents and Injuries, | 3 | Laryngitis or Croup, | 23 |
| Calculus, | 1 | Marasmus, | 1 |
| Cancer of Stomach, | 3 | Old Age, | 7 |
| Consumption, | 33 | Ovarian Tumor, | 1 |
| Convulsions, | 32 | Puerpural Fever, | 11 |
| Diarrhœa, | 8 | Rubeola (Measles), | 2 |
| Diphtheria, | 4 | Rheumatism, | 3 |
| Disease of the Heart, | 3 | Scarlet Fever, | 8 |
| Disease of the Liver, | 1 | Scrofula, | 1 |
| Dropsy, | 8 | Small-Pox, | 17 |
| Erysipelas, | 2 | Spinal Meningitis, | 3 |
| Gangrene, | 1 | Stillborn, | 18 |
| Inflammation of Brain, | 9 | Suicide, | 4 |
| Inflammation of Broncha, | 3 | Typhoid Fever, | 9 |
| Inflammation of Kidneys, | 1 | Unknown, | 8 |
| Inflammation of Liver, | 1 | | |
| Inflammation of Lungs, | 21 | Total, | 256 |
| Inflammation of Stomach & Bowels | 6 | | |

NATIVITIES.

| | | | | | |
|---|---|---|---|---|---|
| Atlantic Ocean, | 1 | England, | 5 | Poland, | 1 |
| Chicago, | 133 | Ireland, | 29 | Colored, | 6 |
| Illinois, | 3 | Scotland, | 1 | Unknown, | 6 |
| Other States, | 31 | France, | 1 | | |
| Canada, | 2 | Germany, | 30 | Total, | 256 |
| Denmark, | 1 | Norway, | 8 | | |

AGES OF THE DECEASED.—Under 5 years, 107; over 5 and under 10 years, 10; over 10 and under 20, 14; over 20 and under 30, 27; over 30 and under 40, 31; over 40 and under 50, 16; over 50 and under 60, 12; over 60 and under 70, 9; over 70 and under 80, 5; over 80 and under 90, 2; stillborn, 18; unknown, 5. Total, 256.

---

NEW HOSPITAL FOR WOMEN AND CHILDREN.—We copy the following from one of our daily papers. Of the mental and professional qualifications of Miss Dr. THOMPSON, we have no personal knowledge; but the boards of consulting physicians, surgeons, and accoucheurs, are composed of reliable and intelligent members of the profession. We hope the institution will be successfully established:—

THE HOSPITAL FOR WOMEN AND CHILDREN.—The hospital for the relief and medical care of poor but respectable women and children has been organized by the election of the necessary officers and a board of trustees. A constitution and by-laws for the proper government of the institution have also been adopted, and the hospital may now be looked on as a fixed fact. The fee for the admission of members has been fixed at five dollars, while the payment of twenty-five dollars entitles a person to be elected a life member of the association. The board of trustees is to consist of forty members, a majority of whom must be ladies. It is also provided that the annual meeting of the association will be held on the last Monday of February in each year. The following are the officers of the society for the current year:

*President*—Mr. J. Y. Scammon.

*Vice-Presidents*—Messrs. J. V. Farwell, and G. S. Bowen.

*Treasurer*—E. I. Tinkham.

*Resident Physician*—Miss Mary H. Thompson.

*Consulting Physicians*—Drs. W. G. Dyas, C. G. Smith, and S. C. Blake.

*Consulting Surgeons*—Drs. A. Fisher, J. Bartlett, and T. D. Fitch.

*Consulting Accouchers*—Drs. Thomas Bevan, H. W. Jones, and E. Marguerat.

*Eye and Ear Surgeon*—Dr. E. L. Holmes.

*Finance Committee*—J. V. Farwell, G. S. Bowen, E. I. Tinkham, C. T. Morse, and F. B. Gardner.

*Executive Committee*—Mrs. W. G. Dyas, Mrs. Colby, Mrs. George Hall, Mrs. W. D. Blain, William Doggett, and T. M. Avery.

*Trustees*—Mr. J. Y. Scammon, Mr. J. V. Farwell, Mr. J. S.

Bowen, Mr. E. I. Tinkham, Rev. Dr. Collyer, Rev. Dr. Tiffany, Mr. W. E. Doggett, Mr. C. Morse, Mr. F. B. Gardner, Mr. T. M. Avery, Mrs. D. P. Livermore, Mrs. H. Sayrs, Mrs. W. Wheeler, Mrs. Dr. Ryder, Mrs. Dr. Tiffany, Mrs. George Hall, Mrs. D. F. Kimble, Mrs. W. Carpenter, Mrs. Dr. S. C. Blake, Mrs. A. Moody, Mrs. Sandford, Mrs. Degenhardt, Mrs. E. A. Kent, Mrs. J. Medill, Mrs. M. C. Scott, Mrs. W. D. Blain, Mrs. W. G. Dyas, Mrs. A. E. Canfield, Miss M. H. Thompson.

---

PERSONAL.—H. A. JOHNSON, A.M., M.D., Prof. of General Pathology and Public Hygiene, in the Chicago Medical College, left home on the fifth of March, on a trip to Europe, where he will probably remain for six or eight months. We cordially wish him a prosperous and pleasant season of travel, and a rapid improvement of health.

---

HOSPITAL APPOINTMENT.—J. S. JEWELL, M.D., Prof. of Anatomy, in the Chicago Medical College, has been appointed one of the Attending Physicians of the Mercy Hospital. This is an excellent appointment in every respect.

---

ILLINOIS STATE MEDICAL SOCIETY.—The following is the list of Committees expected to report at the Annual Meeting of the State Medical Society, to be held in Bloomington, on the first Tuesday in May, 1865:—

*Committee on Practical Medicine and Epidemics:* J. Adams Allen, M.D., of Chicago; C. Goodbrake, M.D., of Clinton; H. Noble, M.D., of Hayworth.

*Committee on Surgery:* D. W. Young, M.D., of Aurora; A. L. McArthur, M.D., of Joliet; L. T. Hewins, M.D., of Oak Alla.

*Committee on Obstetrics:* W. H. Byford, M.D., of Chicago; Lucius Clark, M.D., of Rockford; S. T. Trowbridge, M.D., of Decatur.

*Committee on Drugs and Medicines:* John Bartlett, M.D., of Chicago; F. R. Payne, M.D., of Marshall; T. D. Fitch, M.D., of Chicago.

*Committee on Ophthalmology:* E. L. Holmes, M.D., of Chicago; R. E. McVey, M.D., of Waverly; J. S. Whitmire, M.D., of Metamora; F. B. Haller, M.D., of Vandalia.

*Committee on Spotted Fever:* J. S. Jewell, M.D., of Chicago.

*Committee on Orthopœdic Surgery:* David Prince, M.D., of Jacksonville.

The Committee of Arrangements and the Profession in Bloomington are preparing to give the Society a cordial reception, and we trust the coming meeting will be larger and more profitable than any that have preceded it. We hope the committees will have their reports fully prepared.

---

American Medical Association.—The American Medical Association will hold its Sixteenth Annual Meeting in the City of Boston, on Tuesday, June 6th, 1865.

The following Committees are expected to report:—

On Insanity—Dr. H. R. Storer, Mass., Chairman.

On Exsection and its connection with Conservative Surgery—Dr. Lewis A. Sayres, N. Y., Chairman.

On Drainage and Sewerage of Large Cities, and their Influence on Public Health—Dr. W. J. C. Duhamel, D. C., Chairman.

On Alcohol and its Relations to Man—Dr. G. E. Morgan, Maryland, Chairman.

On Quarantine—Dr. Wilson Jewell, Penn., Chairman.

On Medical Ethics—Dr. John A. Murphy, Ohio, Chairman.

On the Microscope—Dr. James M. Corse, Penn., Chairman.

On the Relations which Electricity sustains to the Causes of Disease—Dr. Squire Littell, Penn., Chairman.

On the Morbid and Therapeutic Effects of Mental and Moral Influences—Dr. A. B. Palmer, Mich., Chairman.

On the Causes of the Extinction of the Aboriginal Races of America—Dr. Geo. V. Suckley, Md., Chairman.

On the Causes and Treatment of Ununited Fractures—Dr. Frank H. Hamilton, N. Y., Chairman.

On Diphtheria—Dr. Lucius Clark, Ill., Chairman.

On the Uses and Abuses of Pessaries—Dr. James P. White, N. Y., Chairman.

On International Medical Ethics—Dr. J. Baxter Upham, Mass., Chairman.

On Climatology and Epidemic Diseases—Dr. C. W. Parsons, R. I., Chairman.

On Autopsies in Relation to Medical Jurisprudence—Dr. T. C. Finnell, N. Y., Chairman.

On so-called Spotted Fever—Dr. James J. Levick, Penn., Chairman.

On the Introduction of Disease by Commerce, and the Means of its Prevention—Dr. A. Nelson Bell, N. Y., Chairman.

On Patent Rights and Medical Men—Dr. David Prince, Ill., Chairman.

On Revision of Plan of Organization—Dr. N. S. Davis, Ill., Chairman.

On Specialists—Dr. J. Homberger, N. Y., Chairman.

On Compulsory Vaccination—Dr. A. N. Bell, N. Y., Chairman.

On Rank of Medical Corps in the Army—Dr. C. S. Tripler, U.S.A., Chairman.

On Rank of Medical Corps in the Navy—Dr. T. L. Smith, N. Y., Chairman.

WM. B. ATKINSON, *Permanent Secretary.*

---

Destruction of Tumors by Electricity.—Mr. Nelaton communicates a paper on the destruction of tumors by an electrolytic method, applicable to cases in which the tumor is deeply seated, and inaccessible to ordinary instruments. The case quoted by him was a naso pharyngian polypus, which was electro-chemically destroyed by inserting two platnam pins into its mass, and making them communicate with the poles of a Bunsen's pill, consisting of nine elements, sixteen centimeters in height by eight in diameter, and arranged for tension. The tumor was destroyed in six sittings, without any effusion of blood, and with very little pain.—*Correspondent of Med. and Surg. Rep.*

---

Army Medical Intelligence—*Promotions.*—Among the promotions in the Medical Department sent to the Senate by the President are the following:—Medical Inspector-General, Joseph K. Barnes, to be Surgeon-General, with the rank of Brigadier-General, August 22d, 1864, *vice* Hammond, dismissed. Surgeon Madison Mills, to be Medical Inspector-General, with the rank of Colonel, December 1, 1864, *vice* Barnes, appointed Surgeon-General.

---

Effect of Petroleum on the Nervous System.—Dr. Georges has observed that the emanations of petroleum have a weakening effect on the muscular system, and cause headache, especially in the case of nervous people, and those who live in a confined atmosphere exposed to these emanations. He states that the latter contain a peculiar principle which may be eliminated, and is found to act principally on the heart and brain. Ether of petroleum may, he adds, be used to cool the teguments during surgical operations, because it causes no pain on the bleeding parts.—*Cor. Med. and Surg. Rep.*

---

Renal Calculi.—Dr. Owen Rees, in the last number of Guy's

Hospital Reports, gives some clinical remarks on calculus diseases. Dr. Rees comments on the common belief that the presence of a calculus in the kidney is always attended with obvious hæmatura; and he cites cases to show that in cases where all the other symptoms of renal calculus are present, there may yet be no blood in the urine. He believes that, in consequence on an undue importance being attached to the absence of this sign, causes of renal calculus have sometimes been treated as if the symptoms were those of gouty or hepatic derangement. Again, Dr. Rees observes, that frequent micturation, though often observed in cases of renal calculus, is not always to be expected. One gentleman of my acquaintance, he says, almost suddenly was seized in the street by violent pain in the side and retraction of the testicles; and on hurrying home passed bloody urine and a calculus, which latter must have been in the kidney many months without producing any other symptom than an uneasy sensation about the loins.

The pain in cases of renal calculus has been said to be more severe on one side than on the other, even when it exists on both sides. But Dr. Rees says, that he has frequently met with cases where the passage of renal calculi has been preceded by all the ordinary symptoms, except pain in the lumbar regions, the discomfort being altogether referred to the sacrum. He also points out a peculiarity which attends the presence of a calculus in the right kidney. The pain, he says, in these cases is referred to the right hypochondrium. It extends downwards towards the umbilicus, but not to the lumbar region. There is a feeling of great distension over the colon, and the bowels are constipated. These are the symptoms so often regarded as significant of biliary calculus, an error easily committed if blood be not perceived in the urine.—*British Medical Journal.*

---

EXISTENCE IN THE HUMAN SUBJECT OF ORGANS UNPROVIDED WITH NERVES, LYMPHATICS, OR CAPILLARIES.—Prof. Simpson, of Edinburgh, in an article in a recent number of the *Medical Times and Gazette* (October 29, 1864,) gives an account of some investigations relative to the structure of the umbilical cord and placenta.

The following are his general conclusions:—

1. The volume of the umbilical cord and fœtal portion of the placenta is formed of nucleated cellular tissue, traversed by the tubes of the umbilical arteries and vein and their numerous placental subdivisions; and the cord and fœtal surface of the placenta are covered by a sheath of serous or seroid membrane.

2. Into the composition of these parts no capillaries, vasa vasorum, lymphatics, nor nerves are found to enter.

3. Hence, in human anatomy, we have these organs, forming a large mass, weighing on an average about two pounds, presenting a type of structure resembling that of some of the inferior zoophites. And,

4. The human mother and her child, two of the most highly-organized beings in existence, are thus temporarily united together, during the intra-uterine life of the latter, by structures of the lowest zoological type.—*Paris Cor. of Lancet.*

---

Deformities of the Feet and the Treatment by Means of Plaster Paris.—Dr. D. C. Enos, in an article read before the Medical Society of the County of Kings, New York, gives the following mode, &c.: Take a straight piece of muslin, wide enough to embrace the head of the tibia, and long enough to extend from the head of the tibia around the heel, and as far as the great toe; then *tear* five or six other pieces of the same size; next stir some plaster of Paris in warm water, till about the consistency of cream; place upon a board, or table, one of the pieces of muslin, and put on it a teaspoonful or two of the plaster, and, with a long knife, spread it evenly over the cloth, so that it shall *be wet and thinly covered;* place upon this another piece of muslin smoothly, and with more of the plaster spread in like manner, and so continue to spread layer after layer until the last, which should *not* be spread; or if so, should be covered with a thin layer of cotton, to come next to the limb. Place the bandage behind the leg, and first bend it around the head of the tibia, and let an assistant hold this firmly, while you next apply it to the sole of the foot as far as the great toe; if it should be too long, turn it back on itself so as to leave the toes exposed; next bend it around so as to neatly embrace the foot to the instep. There is now a redundance of cloth at the angle of the foot, which may be disposed of by folding it on itself so as to smoothly apply the remainder to the ankle and leg; over this a roller should be applied from the toes to the knee, and back again, so that the dry cloth may absorb the moisture from the plaster, and thus facilitate its setting. As soon as the roller is applied, grasp the foot and ankle firmly and forcibly, press the foot towards its normal position, holding it steadily 10 or 15 minutes, till setting has made it firm. Sometimes he embraces the foot and ankle; first, by taking a piece of muslin, from one or two yards long, and from six to eight inches wide, spread it thinly with the plaster, double it longitudinarily and spread it again, and double it once more, and apply it to the foot and ankle, somewhat like an ordinary roller, holding the foot as before till the plaster sets. This answers for deformities of the foot, simply, where its position with the tibia is normal; but if it is abnormal, as it usually is, the retentive apparatus must extend to the knee, after embracing the foot, as already fixed. Sometimes this can be advantageously done by putting a number of folds of the plastered muslin along on the top of the foot and anterior part of the leg, strengthening it in the instep by an extra quantity of plastered cloth, binding this on with a roller, and holding the foot firmly up till the plaster sets. Occasionally, to strengthen the first described application, he applied over it this plaster and four folded roller around the foot and ankle, thus making a figure of eight bandage. After the apparatus had been on for three or four days, it can be removed, and a new one applied, when, from the absorption that has taken place, an increased amount of pressure can be borne, and the foot made to approach still more towards its normal position.—*Buffalo Medical and Surgical Journal.*

